# DEC-205 receptor-mediated long-circling nanoliposome as an antigen and *Eucommia ulmoides* polysaccharide delivery system enhances the immune response via facilitating dendritic cells maturation

**DOI:** 10.1080/10717544.2020.1844343

**Published:** 2020-11-10

**Authors:** Haibo Feng, Xiaonong Yang, Jing Fan, Linzi Zhang, Qianqian Liu, Dongkun Chai

**Affiliations:** a College of Animal Husbandry and Veterinary Medicine, Southwest Minzu University, Chengdu, P. R. China; b Key Laboratory of Ministry of Education and Sichuan Province for Qinghai-Tibetan Plateau Animal Genetic Resource Reservation and Utilization, Chengdu, P. R. China; c Sichuan Industrial Institute of Antibiotics, Chengdu University, Chengdu, P. R. China; d Department of Veterinary Medicine, Southwest University, Rongchang, P. R. China

**Keywords:** DEC-205 receptor, *Eucommia ulmoides* polysaccharide, nanoliposome, dendritic cells, immune response

## Abstract

DEC-205 receptor-mediated dendritic cells (DC) targeting nanoliposomes is a promising delivery system in eliciting an immune response against pathogens. When this delivery system carries both antigen and immunomodulator, it can effectively regulate the DC function as well as the initial T cell response. To maximize the desired therapeutic effects of *Eucommia ulmoides* Oliv. polysaccharides (EUPS), and induce an efficient humoral and cellular immune response against an antigen, we encapsulated the OVA and EUPS in long-circling nanoliposomes and conjugated it with anti-DEC-205 receptor antibody to obtain a DEC-205-targeted nanoliposomes (anti-DEC-205-EUPS-OVA-LPSM). The physicochemical properties and immune-modulating effects were investigated *in vitro* and *in vivo* by a series of the experiment to evaluate the targeting efficiency of anti-DEC-205-EUPS-OVA-LPSM. *In vitro,* anti-DEC-205-EUPS-OVA-LPSM (160 μg mL^−1^) could enhance DCs proliferation and increase their phagocytic efficiency. *In vivo* anti-DEC-205-EUPS-OVA-LPSM remarkably promoted the OVA-specific IgG and IgG isotypes levels, enhanced the splenocyte proliferation, and induced the NK cell and CTL cytotoxicity. Besides, the anti-DEC-205-EUPS-OVA-LPSM enhanced the maturation of DCs. These findings suggest that the DEC-205 receptor antibody-conjugated EUPS nanoliposome can act as an efficient antigen delivery system to enhance the cellular and humoral immune response by promoting DC maturation. This indicates that the anti-DEC-205-EUPS-OVA-LPSM has significant potential as an immune-enhancing agent and antigen delivery system.

## 1. Introduction

Animal husbandry incurs a heavy loss worldwide due to infectious diseases, notably viral infections. Vaccination remains the most effective means to combat infectious diseases. Vaccination regimes are strictly followed in the farms; even then, in most of the cases, animals do not produce the desired immune response due to which most of the infections could not be completely controlled (Monath, 2013; Afolabi et al., 2019; Fan et al., 2020; Ma et al., 2020). Application of appropriate immune enhancers can improve the vaccine efficacy and assist the vaccines in producing better immune responses (Bookstaver et al., 2018; Ratnapriya et al., 2019). The application of immune enhancers has become one of the crucial tools in improving vaccine-induced immune responses. Various researchers have demonstrated that traditional Chinese medicine and its effective components can significantly improve the vaccine-induced cellular and humoral immune responses. A variety of traditional Chinese medicine and its core components as immune enhancers of vaccines are currently being used in animals and humans (Rodrigues Barbosa et al., 2020).

Over the past few years, various studies have demonstrated the adjuvant effect of a myriad of traditional Chinese medicinal plant polysaccharides on the antigen-specific humoral and cellular immune responses in pathogenic infections (Feng et al., 2013, 2015; Sun et al., 2018). Plant polysaccharides have several advantages over other adjuvants, such as better safety and tolerability, and easy to manufacture formulation. Thus, the plant polysaccharides have the potential of being used as adjuvants for the vaccines against infectious agents and malignancies. The *Eucommia ulmoides* Oliv. (EU) is Chinese traditional medicine with multiple pharmacological activities. In our previous study, we have found that the polysaccharides extracted from *E. ulmoides* Oliv. (EUPS) regulated DCs maturation and served as an immune-enhancer; besides, it activated the DCs, and promoted the antigen-specific humoral and cellular response (Feng et al., 2016).

Dendritic cells (DCs) as an antigen-presenting cell (APC), can integrate innate and adaptive immune responses. The bone marrow hematopoietic progenitors derived immature DCs circulate in the blood and can be spotted in tissues as sentinel cells, and recognize antigen or take up the pathogen from the extracellular environment by employing the receptor-mediated endocytosis, pinocytosis, and micropinocytosis (Sadeghzade et al., 2020; Shortman, 2020). It can efficiently process distinct antigens and load them to major histocompatibility complexes (MHC I and MHC II). DCs eventually migrate to the secondary lymphoid organ (lymph nodes). They transform to mature DCs and present the processed antigens to the Th cells, thus initiating the adaptive immune response after being recognized by CD4^+^ or CD8^+^ T cells (Patente et al., 2019). DCs initiate cellular signaling. However, this signal is insufficient to induce T cell activation and necessitates a secondary signal (Giovanelli et al., 2019; Zhu et al., 2019). The costimulatory signaling molecules such as CD40, CD80, and CD86 are expressed on the DCs surface, which generates the secondary signal. These receptors interact with CD40L and CD28 on the T cell surface, activate T cells, and elicits antigen-specific immune responses. This indicates that the successful delivery of antigens by DCs is a crucial factor for effective immunization against infectious diseases, cancer, and autoimmune diseases (Qian & Cao, 2018). Therefore, multiple studies are investigating the means to specifically target the antigens to the immature DCs by using appropriate carriers and delivery routes. Immature DCs take up antigens in three ways: (1) pinocytosis, where DCs swallow the liquid, high molecular material, and some small nanoparticles, (2) endocytosis, where DCs endocytose the antigen or carrier containing specific ligand-mediated by receptor, (3) phagocytosis, where DCs phagocytose the large nanoparticles and microparticles (Rueda et al., 2017; Peron et al., 2018). In recent years, DCs mediated immunotherapy has been widely used in *in vitro* experiments and animal models because of its diversity, plasticity, and special status in the immune response process (Peron et al., 2018). However, the diversity of DCs, *in vivo,* is minimal, and only a small part of exogenous drugs or antigens are presented by DCs. Therefore, it is crucial to improve the targeting of drugs or exogenous antigens to dendritic cells and the bioavailability, to reduce the dosage of antigen protein or drugs and the cost of vaccines, and to promote the immune response (Belz et al., 2004; Rueda et al., 2017).

Vectors targeting the dendritic cell receptors is a commonly used method of delivering drugs or antigens to DCs. DEC-205, a member of the C-type lectin macrophages mannose receptor family, is predominantly expressed on DCs in the T cell area of lymph nodes (Lewis et al., 2012). DEC-205 is expressed by the CD8^+^ subset of the DCs, which assists in the cross-presentation of Antigen. The cytoplasmic region of the DEC-205 contains protein motifs that mediate endocytosis and target late endosomes/MHC II cells. It makes the DEC-205 more efficient in loading antigens to MHC II molecules and later presenting them to T lymphocytes (Jáuregui-Zúñiga et al., 2017). DEC-205 targeted vaccination can effectively enhance vaccine efficacy as it can significantly increase both CD4 and CD8 T cell responses (Bandyopadhyay et al., 2011). Currently, multiple studies are focusing on the cross-linking of antigens to the DEC-205 monoclonal antibody to achieve antigen targeting. However, the cross-linked compounds can readily interact with proteases and immune systems, *in vitro*, and the experimental results of the model are ideal, but the *in vivo* effect is poor, and the bioavailability of drugs is low (Cruz et al., 2014).

The dendritic cells targeted carrier, depending upon its size, can be easily absorbed by the DCs than the carrier microspheres, nanoparticles, liposomes, and other particles encapsulated or linked free antigens. Liposomes serve as multifaceted carriers, which can deliver drug and biologically active molecules. The advantage of liposomes is their large carrying capacity and slow-release; besides, liposomes reduce drug dosage, promote the APC’s antigen uptake, and so on (Wang et al., 2019; Irvine & Read, 2020). The antigens and mature regulator of DCs can be encapsulated together in the same liposome to control the outcome of the immune response. Additionally, the general polypeptide vaccine cannot induce a strong CTL response. Still, it can enter the MHC I molecular antigen presentation process after being encapsulated in a liposome, to induce humoral and cellular immunity at the same time (Crommelin et al., 2020).

We encapsulated the antigen and EUPS in nanoliposomes, and designed a DEC-205-targeted nanoliposomes to dendritic cells (DCs) to maximize the desired therapeutic effects of EUPS, and induce an efficient humoral and cellular immune response in response to antigen. In the present study, *E. ulmoides* polysaccharide and ovalbumin nanoliposome (EUPS-OVA-LPSM) were prepared by membrane dispersion method. The anti-DEC-205-EUPS-OVA-LPSM was prepared by conjugating the DEC-205 antibody to the EUPS-OVA-LPSM nanoliposome. The physicochemical property and the targeting effect of the anti-DEC-205-EUPS-OVA-LPSM nanoliposome were investigated by determining their effect on the humoral and cellular immune response *in vivo*. We found that the DEC-205 antibody conjugated nanoliposome could significantly enhance the cellular and humoral immune responses against OVA in the mice.

## 2. Materials and methods

### 2.1. Reagents

The phospholipid, cholesterol, distearoyl phosphoethanolamine-PEG2000 (DSPE-PEG2000PDP), DSPC, SPDP crosslinker, FITC-dextran, concanavalin A (ConA), and lipopolysaccharide (LPS) were procured from Sigma Chemical Co. (Saint Louis, Missouri, USA). Purified EUPS with a purity of ≥97% was procured from Tianqi Biotechnology Co., Ltd. (Shanxi, China). A standard high-performance liquid chromatography-refractive index detection method was utilized to purify the polysaccharide preparation (≥97% purity). Rat anti-mouse DEC-205 monoclonal antibody was purchased from Thermo Fisher Scientific Co., Ltd. (Dallas, TX). RPMI-1640, fetal bovine serum, were procured from Life Technologies Corporation (Carlsbad, CA). Cell Counting Kit-8 (CCK-8) was obtained from the Beyotime Institute of Biotechnology (Haimen, Jiangsu, China). Fluorescently-labeled anti-mouse monoclonal antibodies (CD80-PE, CD40-FITC, CD86-PE, MHC-II-PE, and CD3-FITC, CD8-APC, CD4-PE) were purchased from eBioscience (San Diego, CA).

### 2.2. Preparation of anti-DEC-205-EUPS-OVA-LPSM

#### 2.2.1. Preparation of drug-loaded long-circulating liposomes

The mixture of DSPC, cholesterol, distearoyl phosphoethanolamine-PEG (2000) amine and DSPE-PEG (2000) PDP (molar ratio 45:30:2:0.4) was dissolved in chloroform was added to a small beaker. To fully dissolve the mixture, it was shaken for 15 min by utilizing the ultrasonic instrument (Alam et al., 2017; Chaubet et al., 2019). The mixed solution was transferred to the rotary evaporation flask, and the flask was rotated at 38 °C. The solution was evaporated under reduced pressure to eliminate chloroform from the solution and create a dry film on the inner wall of the flask. EUPS (2.5 mg/mL) and OVA (1.25 mg/mL) were dissolved in distilled water and transferred to the rotary evaporating round bottle with a dry film. It was continuously spun under the reduced pressure at 38 °C until the dry film in the bottle was removed. When the resulting liquid in the bottle was evenly colored, and the bottle wall was bright, the rotation of the flask was stopped and continually hydrated for about 10 min. The solution was collected and passed through 0.45 μm as well as 0.22 μm microporous membrane, in turn, followed by the addition of the drug to obtain the liposomes (Allen et al., 1995).

#### 2.2.2. Preparation of the anti-DEC-205-EUPS-OVA-LPSM

The heterobifunctional reagent N-Succinimidyl 3-(2-pyridyl dithio)propionate (SPDP) was employed to cross-link the DEC-205 antibody and liposomes (Hu et al., 2008). PDP-IgG was synthesized by mixing the 5 mg/mL of anti-mouse DEC-205 antibody or the control IgG with 6.25 mg/mL of SPDP solution and reacting the mixture at 23 ∼ 25 °C for 30 min. Furthermore, the Sephadex G-25 column was used to remove the excessive SPDP and other reaction by-products from the reaction solution, the equilibrium and elute solution was acetic acid buffer with a concentration of 100 mmol/L, pH 4.5. Later, part of the PDP-IgG was harvested, and the dithiothreitol (DTT) was added to attain the final concentration of DTT as 50 mmol/L. This mixture was reacted at 23–25 °C for 25 min with continuous stirring. Excessive DTT in the reaction mixture was removed by passing through the Sephadex G-25 column (equilibrium and elution buffer concentration were 100 mmol/LPBs, pH 7.5). The part of IgG-SH was collected and mixed with liposome immediately in the molar ratio of 1:1000. The mixture was stirred in the dark at room temperature for 18 h. Unconjugated IgG-SH was removed by the Sepharose CL-4B column, and the long-circulating immune liposome (OVA-EUPS-iLPSM) was combined with DEC-205 antibody.

#### 2.2.3. Characterization of the anti-DEC-205-EUPS-OVA-LPSM by transmission electron microscope (TEM)

The liposome samples were diluted ten times with PBS, placed on the slide, and stained with the negative dye-1% phosphotungstic acid for 2 min. Slides were later placed on the special copper net, dried for 2 min to concentrate the particles, and deposit them on the copper net. It was later examined under the TEM (model H-7650, Hitachi, high technologies, Japan), and the images were captured.

#### 2.2.4. Particle size analysis

Laser particle size analyzer (Anton Paar, Litesizer 500, Austria) evaluated the particle size, zeta potential, and the colloidal dispersion system stability of the nanoliposomes.

#### 2.2.5. The leakage rate of liposome stored in different conditions

The dialysis membrane method determined the leakage rate of liposome (Wallenwein et al., 2019). Concisely, the liposomes were dialyzed in PBS buffer at 4 °C and 25 °C with continuous stirring for 14 days. The nanoliposomes particle size was examined every alternate day and supplemented with 1 mL PBS simultaneously. On day 14, the dialysis bag was cut, and the liposomes solution and dialysate were mixed. This was followed by the addition of 1% Triton X-100, and the mixture was stirred continuously. The fluorescence spectrophotometer measured the fluorescence at 485 nm (excitation wavelength) and 515 nm (emission wavelength), and the value was recorded as *A* ∞ (drug leakage amount at an infinite time). The fluorescence value of the samples at each time point was recorded as *At* (FITC dextran leakage of the samples at time *t*). The leakage rate of liposomes was calculated with the following equation: Leakage rate (%) = *At*/*A* ∞.

#### 2.2.6. The particle size determination of liposomes stored in different conditions

The anti-DEC-205-EUPS-OVA-LPSM liposomes were stored at 4 °C and 25 °C for 14 days. The particle size was determined by particle size analyzer on days 2, 4, 6, 8, 10, 12, and 14. The particle size of the liposomes samples taken at each time point was recorded.

### 2.3. Experiment in vitro

#### 2.3.1. Cytotoxicity of anti-DEC-205-EUPS-OVA-LPSM on DCs

The DCs single cells solution was prepared according to our previous report (Jáuregui-Zúñiga et al., 2017) The DC suspension was adjusted to 1 × 10^6^/mL and seeded onto a 96-well plate. Once the DCs grew into monolayers, 100 μL of different concentrations (5, 10, 20, 40, 80, 160, 320, 640, 1280 μg/mL) of anti-DEC-205-EUPS-OVA-LPSM solution were added to the wells, and cultured for a further 24 h. Next, 10 μL of Cell Counting Kit-8 (CCK-8) reagent was added to each well and the OD was measured using a microplate reader at 450 nm after 4 h.

#### 2.3.2. Phagocytosis of anti-DEC-205-EUPS-OVA-LPSM by DC cells

The DCs solution was inoculated in a 6-well cell-culture plate with a round coverslip. After 24 h of culture, FITC-OVA, anti-DEC-205-EUPS-OVA-LPSM were added, after a further incubation of 12 h, and the slides were taken out and fixed with 4% paraformaldehyde for 20 min and permeabilized with Triton X-100 for 4 min. Next, they were stained with Phalloidin-iFluor 555 Reagent for 50 min and with DAPI staining solution for 5 min. Lastly, the DCs were mounted with 90% glycerol and photographed using a confocal laser scanning microscope (LSM 800, ZEISS, Germany).

#### 2.3.3. The delivery effect of anti-DEC-205-EUPS-OVA-LPSM on OVA

The DCs were inoculated in a 6-well cell-culture plate with a round coverslip. After 24 h of culture, FITC-OVA, anti-DEC-205-EUPS-OVA-LPSM was added, and cultured at 37 °C for 12 h, then all supernatant was absorbed and discarded, PBS was washed three times, and 100 μL SDS cell lysate was added into each well to digest the cells. The fluorescence intensity (excitation wavelength 495 nm, emission wavelength 528 nm) was measured by a fluorescent enzyme labeling instruments.

#### 2.3.4. Cytokine production by DCs in vitro

The single DCs suspension (2.5 × 10^6^ cells/mL) were seeded to a 96-well plate, and anti-DEC-205-EUPS-OVA-LPSM (160 μg/mL) was added, and the plates were incubated for 72 h. the supernatant was harvested and the concentration of IL-12 was detected by ELISA kit (Thermo Fisher Scientific Inc, Minneapolis, MN) according to the manufacturer’s instructions.

### 2.4. Experiment in vivo

#### 2.4.1. Animals and immunization

Females ICR mice (age: 5 weeks, Grade II, weight: 18–22 g) were provided by Sichuan Laboratory Animal Center, (China). All animal procedures were performed as per internationally accepted principles, mentioned in the government of China issued Guidelines for Keeping Experimental Animals and approved by the IACUC, Southwest University. Eight groups containing ten female ICR mice in each group were formed by the random division. These eight groups were anti-DEC-205-EUPS-OVA nanoparticles group, naive group, EUPS group, OVA group, OVA plus EUPS group, blank LPSM group, OVA-EUPS-nanoparticles group, and OVA plus alum group. Mice were immunized on day 0 and 14 by administering the following vaccine formulations subcutaneously in the hind limb: 200 μL anti-DEC-205-EUPS-OVA nanoparticles (OVA 50 μg, EUPS 100 μg), 200 μL saline, 0.1 mg EUPS alone, 200 μL OVA (50 μg), 200 μL OVA (50 μg) plus100 μg EUPS, 0.5 mg blank LPSM, 0.5 mg OVA-EUPS-nanoparticles (OVA 50 μg, EUPS 100 μg), 200 μL OVA (OVA 50 μg) plus 200 μg alum.

#### 2.4.2. ELISA

Serum samples were taken 2 weeks post-second booster dose to evaluate the OVA-specific IgG, IgG1, IgG2b, and IgG2a levels. Indirect ELISA was employed to measure the specific IgG titer and isotype levels, as described previously (Feng et al., 2013). 100 μL per well OVA solution was added to the 96-wells microtiter plate (Costar) to coat the wells with the OVA solution. These plates were incubated at 4 °C for 20 h. It was followed by three consecutive washes and the addition of the 100 μL per well-skimmed milk (5%, w/v), followed by incubation at 37 °C for 1 h. Furthermore, diluted serum samples (1:50) were added to the 96-plate (100 μL/well) followed by incubation at 37 °C for 1 h, and washed thrice. The HRP-conjugated secondary antibodies (IgG, IgG1, IgG2b, and IgG2a) were added to each well and incubated at 37 °C for 1 h. Each well was washed thrice, and substrate solution (Beyotime Biotechnology, Jiangsu, China) was added to each well followed by incubation at 37 °C for 10 min. The reaction was halted by 50 μL of 2 M H_2_SO_4_ supplementation. The optical density (OD) was measured at 450 nm by an ELISA reader (Model 680, Bio-Rad, CA).

#### 2.4.3. Splenocyte proliferation assay

The splenocyte proliferation assay was conducted, as mentioned in the previous study (Feng et al., 2016). CCK-8 assay evaluated the T cell proliferation, as per the manufacturer’s instructions. The data are expressed as a stimulation index (SI), and SI was calculated by using the following equation: SI = OD (antigen-stimulated cultures)/OD (unstimulated cultures).

#### 2.4.4. Analysis of the concentration of serum cytokines

Blood samples were collected 24 h after the last anti-DEC-205-EUPS-OVA-LPSM administration, and it was allowed to clot for 2 h. The serum was separated from the blood clot and stored at −20 °C until further use. The cytokines (IL-4 and IFN-γ) levels were estimated in the serum utilizing the ELISA kits as per the manufacturer’s instructions.

#### 2.4.5. In vivo CTL assay

An *in vivo* CTL assay was conducted as per our previous protocol (Feng et al., 2015). Briefly, on day 14, following booster immunization, target and control cells in 1:1 ratio were injected via tail vein into the immunized mice (Total cells: 2 × 10^7^ cells/mouse). After 4 h, the mice spleen was removed, and the splenocytes were isolated and analyzed by flow cytometry (FACS Calibur, BD Biosciences, East Rutherford, NJ). Specific lysis was determined by employing the following equation: Lysis ratio = Percentage CFSE^low^/Percentage CFSE^high^, Percentage specific lysis = [1 − (Unprimed ratio/Primed ratio) × 100].

#### 2.4.6. Natural killer (NK) cell cytotoxicity assay

NK cell cytotoxicity was assessed as described previously (Feng et al., 2013, 2015). The cytocidal activity of the CFSE + PI labeled cells was analyzed by flow cytometry.

#### 2.4.7. T-Lymphocyte phenotyping by flow cytometry

Peripheral blood samples or single-cell splenocyte suspensions were prepared as described previously (Zhao et al., 2015). Briefly, splenocyte suspensions (1 × 10^6^ cells/mL) were stained with either anti-CD3-FITC, anti-CD8-APC, or anti-CD4-PE antibodies (10 μL) at 4 °C for 1 h. After washing twice, the cells were fixed by 1% PFA (paraformaldehyde) and evaluated with a FACS Calibur (BD Biosciences). The proportion of CD4+, CD3+, and CD8+ T lymphocytes in the mice peripheral blood were examined by using the Cell Quest Pro software (BD Biosciences). The outcomes are demonstrated as the of CD4+ (%) and CD8+ (%).

### 2.5. Statistical data analysis

Data were analyzed with SPSS software, Version 11.5 from SPSS Inc. (Chicago, IL). For multiple comparisons between groups, ANOVA with Bonferroni post-hoc test was used. Results are expressed as the mean ± standard deviation (SD) of the mean. The values with *p* < .05 were statistically significant and depicted by a single asterisk in the figures.

## 3. Results

### 3.1. The physicochemical properties of the anti-DEC-205-OVA-EUPS-LPSM

#### 3.1.1. The ultra micro-structure characterization of the anti-DEC-205-OVA-EUPS-LPSM

**Figure 1.**
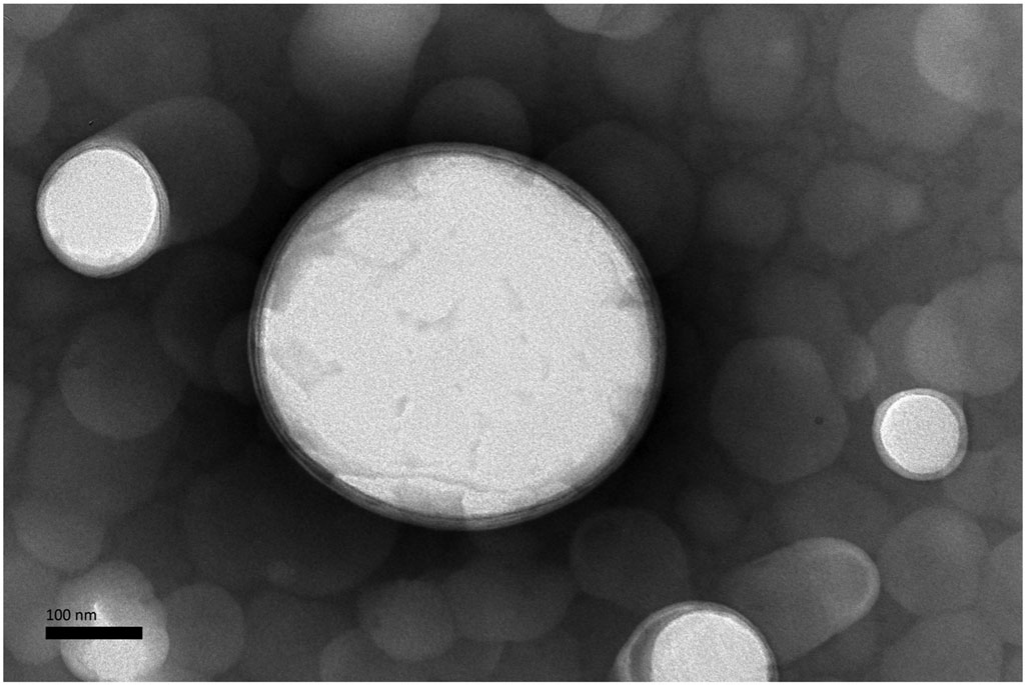
Transmission electron microscope (TEM) micrographs of the anti-DEC-205-OVA-EUPS-LPSM, SMP (50,000×).

The TEM findings indicated the morphological similarity of the anti-DEC-205-OVA-EUPS-LPSM, where both the liposomal entities appeared as the spherical or nearly spherical structure with the double membrane (Figure 1). The LPSM showed an average particle size of about 200 nm.

#### 3.1.2. The effect of temperature and storage time on leakage rate of the anti-DEC-205-OVA-EUPS-LPSM

**Figure 2.**
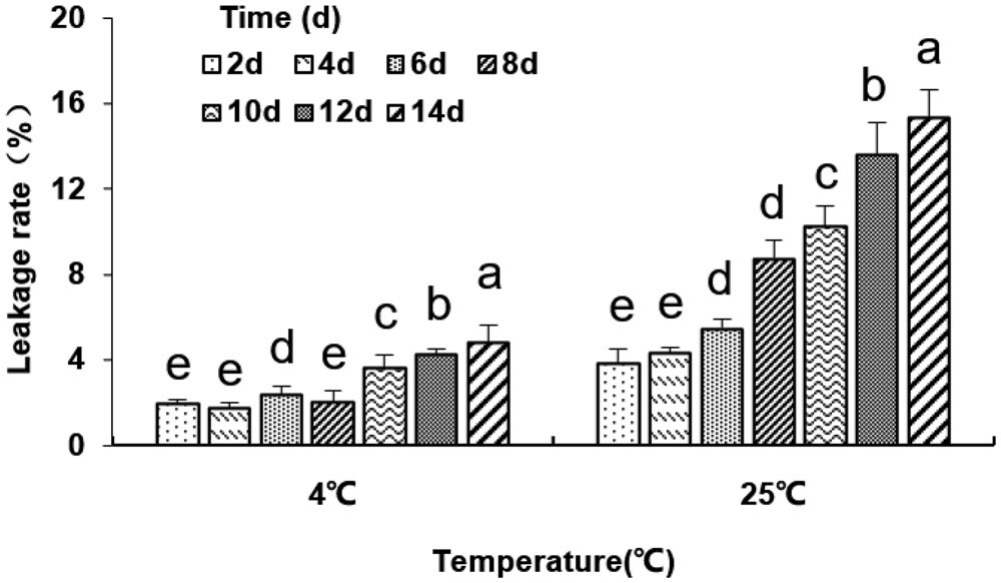
The leakage rate (%) of the anti-DEC-205-OVA-EUPS-LPSM at 4 °C and 25 °C in 14 days. The liposomes dialysis in PBS buffer at 4 °C and 25 °C under the stirring condition for 14 days. The nanoliposomes particle size was determined on days 2, 4, 6, 8, 10, 12, and 14. Results are presented as the mean ± SD (*n* = 4), Bars marked with different letters (a-e) indicate the statistically significant differences (*p* < .05).

The dialysis membrane method was used to determine the leakage rate of liposomes (Hu et al., 2008). The outcome showed that at the storage temperature of 4 °C, the cumulative leakage rate of liposomes was less than 2% in 4 days and less than 5% in 14 days. However, the leakage rate of liposomes was significantly higher when stored at 25 °C (*p* < .05, Figure 2), which was maybe due to the enhanced membrane fluidity and the depleted aggregation stability of liposomes. Besides, antibodies on the liposome surface led to increased interaction between the liposomes and reduced liposomal membrane stability. This suggests that liposomes should be preserved at low temperatures (4 °C).

#### 3.1.3. The effect of temperature and storage time on the particle size of the anti-DEC-205-OVA-EUPS-LPSM

**Figure 3.**
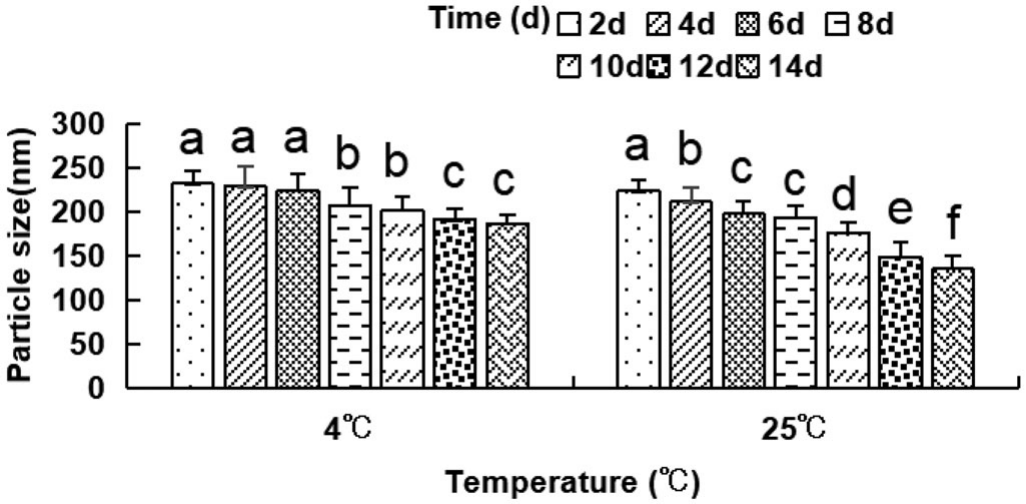
The particle size of the anti-DEC-205-OVA-EUPS-LPSM at 4 °C or 25 °C in 14 days. The liposomes were stored at 4 °C or 25 °C for 14 days. The particle size was determined on days 2, 4, 6, 8, 10, 12, and 14 by laser particle size analyzer. Results are presented as the mean ± SD (*n* = 4), Bars marked with different letters (a–e) indicate the statistically significant differences (*p* < .05).

The dynamic laser light scattering evaluated the particle size distribution, every alternate day for a total of 14 days at 4 °C and 25 °C. The outcomes indicated that the particle size of liposomes significantly decreased on days 8 and 10 as compared to day 2–6 at 4 °C, as depicted in Figure 3. The size of liposomes reduced considerably on days 12–14 as compared to days 2–8 at 4 °C and on days 4 and 6 as compared to day 2 at 4 °C. The particle size of liposomes on days 8–14 also was found to be remarkably reduced as compared to days 2–6 at 25 °C. These findings suggest that the particle size of the anti-DEC-205-OVA-EUPS-LPSM gradually decreased with an increase in storage time at 4 °C or 25 °C. Moreover, the particle size of liposomes stored at 25 °C reduced significantly than the liposomes stored at 4 °C. The decrease in particle size might be due to drug leakage.

### 3.2. In vitro tests

#### 3.2.1. The cytotoxicity analysis of anti-DEC-205-EUPS-OVA-LPSM on DCs

**Figure 4.**
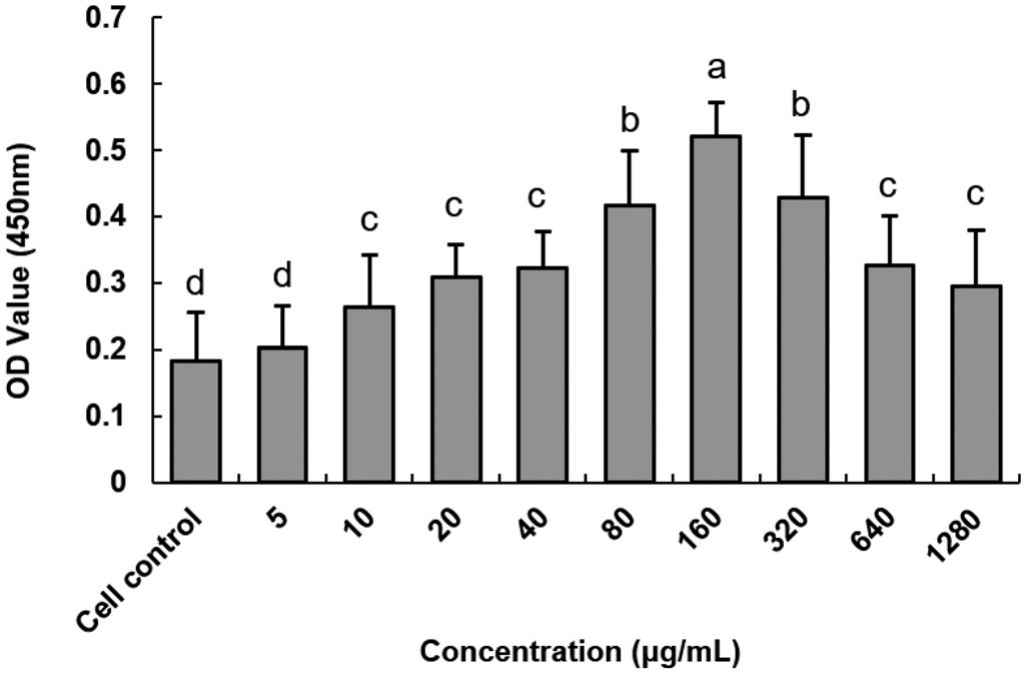
Effect of anti-DEC-205-EUPS-OVA-LPSM on DCs activity. cell activity was measured using the CCK-8 method. Results are presented as mean ± SD (*n* = 6). Bars marked with different letters (a–e) indicate the statistically significant differences (*p* < .05).

The *Cytotoxicity* of anti-DEC-205-EUPS-OVA-LPSM on DCs was illustrated in Figure 4. The anti-DEC-205-EUPS-OVA-LPSM was not cytotoxic to DCs in the concentration range of 5–1280 μg mL^−1^ and even increased cell proliferation. Maximum cell proliferation activity was observed at a concentration of 160 μg mL^−1^ and was significantly different from the blank control group (*p* < .05). Therefore, a concentration of 160 μg mL^−1^ was selected for subsequent experiments.

#### 3.2.2. Effects of anti-DEC-205-EUPS-OVA-LPSM on the phagocytic activity of DCs

**Figure 5.**
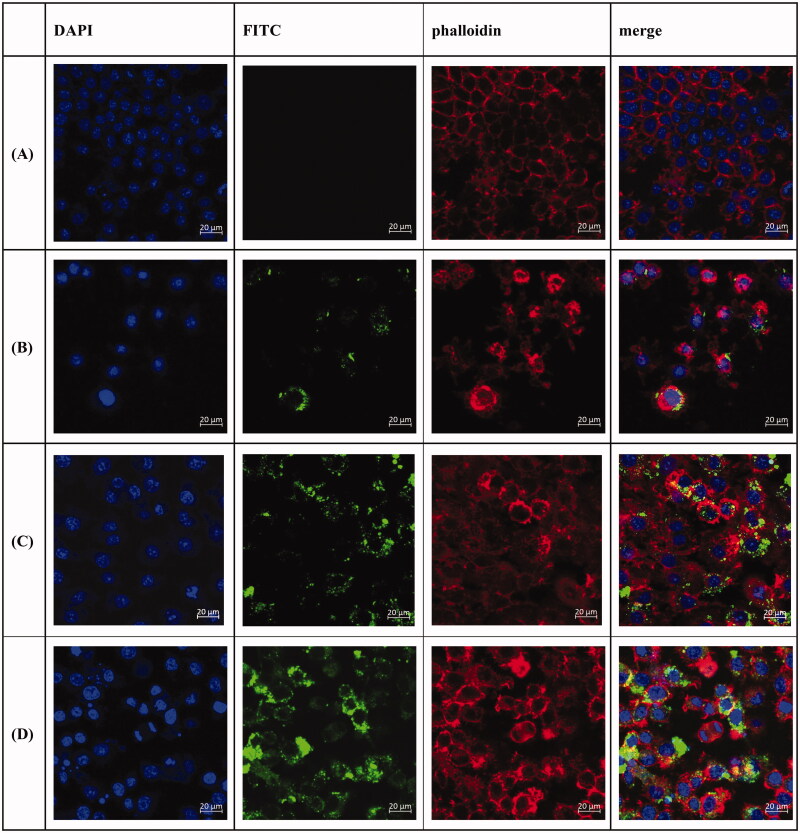
Laser confocal scanning microscopy of anti-DEC-205-EUPS-OVA-LPSM uptake by DCs. DCs were inoculated in a 6-well cell-culture plate with a round coverslip. After 24 h of culture, OVA, and anti-DEC-205-EUPS-OVA-LPSM were added separately. After incubation for 12 h, the slides were taken out and fixed and stained using DAPI and Phalloidin-iFluor 555. Blue fluorescence is the nucleus labeled by DAPI, while red fluorescence indicates the actin stained with Phalloidin-iFluor 555. Cells were mounted with 90% glycerol and photographed using a confocal laser scanning microscope. (A) Control, (B) OVA, (C) EUPS-OVA-LPSM, and (D) anti-DEC-205-EUPS-OVA-LPSM.

In order to explore the ability of DCs to take up anti-DEC-205-EUPS-OVA-LPSM, the phagocytic effect of DCs on antigens was observed using laser confocal scanning microscopy. Different fluorescence intensities indicate the uptake of different antigens by DCs. It can be seen in Figure 5 that the fluorescence intensities of the anti-DEC-205-EUPS-OVA-LPSM and EUPS-OVA-PLGA-LPSM groups are significantly higher than that of the OVA group. This shows that EUPS could enhance the ability of the DCs to take up antigens. Compared to the EUPS-OVA-PLGA-LPSM group, the fluorescence intensity of the anti-DEC-205-EUPS-OVA-LPSM group was significantly increased. We found that anti-DEC-205-EUPS-OVA-LPSM was mainly distributed in the cytoplasm and there was also a large amount of antigen adsorbed on the cell surface to be phagocytosed by DCs. These findings indicated that the anti-DEC-205 antibody conjugated the LPSM could enhance DCs to take up more OVA.

#### 3.2.3. The delivery effect of anti-DEC-205-EUPS-OVA-LPSM on OVA

**Figure 6.**
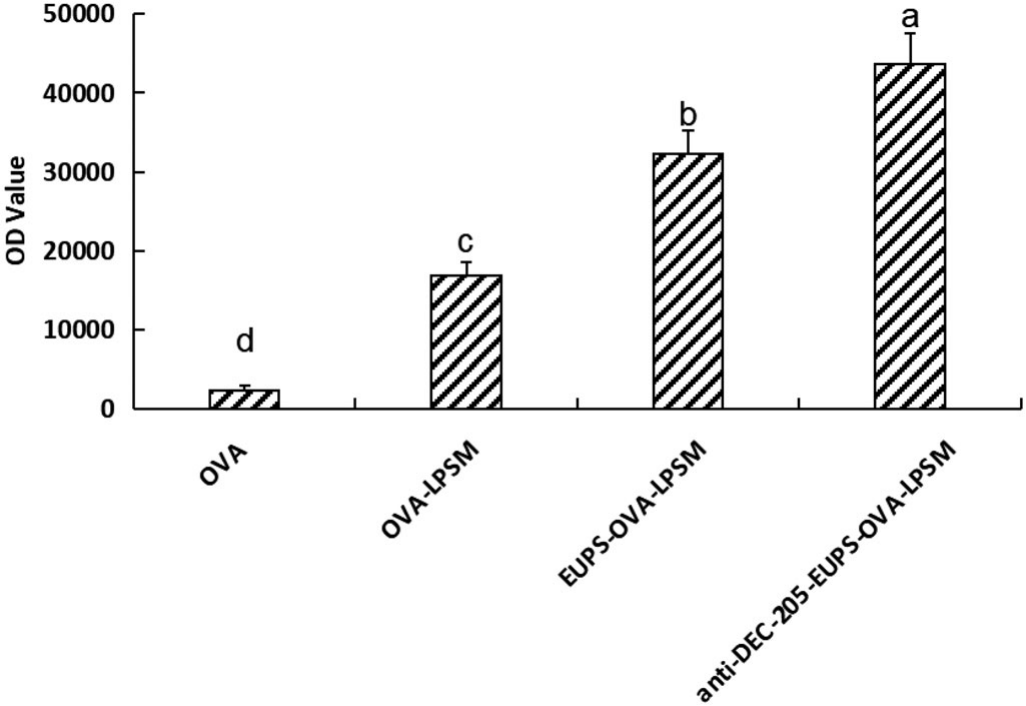
Phagocytic efficiency of different nanoliposome on DCs. Results are presented as mean ± SD (*n* = 6). Significant differences are designated as different letters (a–d) (*p* < .05).

As shown in Figure 6, The fluorescence intensity in anti-DEC-205-EUPS-OVA-LPSM, EUPS-OVA-LPSM, and OVA-LPSM significantly higher than the free OVA group (*p* < .05). In the present study, the fluorescence intensity in the anti-DEC-205-EUPS-OVA-LPSM group was significantly higher than that of the EUPS-OVA-LPSM group (*p* < .05). Indicating that the phagocytic effect of anti-DEC-205-EUPS-OVA-LPSM group was significantly higher than that of EUPS-OVA-LPSM group. The anti-DEC-205 antibody conjugated nanoliposome can improve phagocytosis efficiency than those of nanoliposome. The anti-DEC-205 antibody conjugated nanoliposome can make the nanoliposome have active targeting and significantly improve the phagocytosis rate of the nanoliposome.

#### 3.2.4. Effects of anti-DEC-205-EUPS-OVA-LPSM on the production of IL-12 by DCs

**Figure 7.**
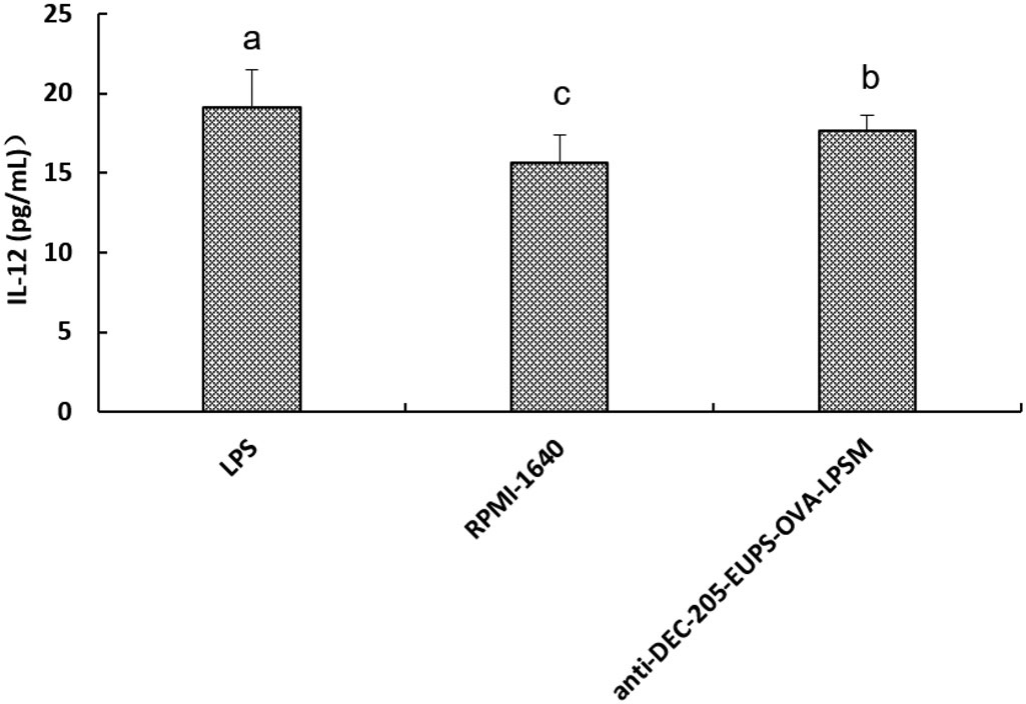
Effects of the anti-DEC-205-EUPS-OVA-LPSM on IL-12 production by DCs *in vitro*. DCs were isolated from ICR mice and incubated with anti-DEC-205-EUPS-OVA-LPSM (160 μg/mL), LPS, or RPMI-1640. The concentrations of IL-12 in supernatants were determined by ELISA. The values are presented as mean ± SD. Significant differences are designated as different letters (a–c) (*p* < .05).

As shown in Figure 7, When DCs were treated with anti-DEC-205-EUPS-OVA-LPSM (160 μg/mL), the concentration of IL-12 in the supernatant was significantly higher than in RPMI1640-treated cells (*p* < .05). The data suggested that anti-DEC-205-EUPS-OVA-LPSM can significantly stimulate DCs to secret the IL-12.

### 3.3. In vivo tests

#### 3.3.1. Effects of the anti-DEC-205-EUPS-OVA-LPSM on the OVA-Specific IgG and the IgG subclasses

**Figure 8.**
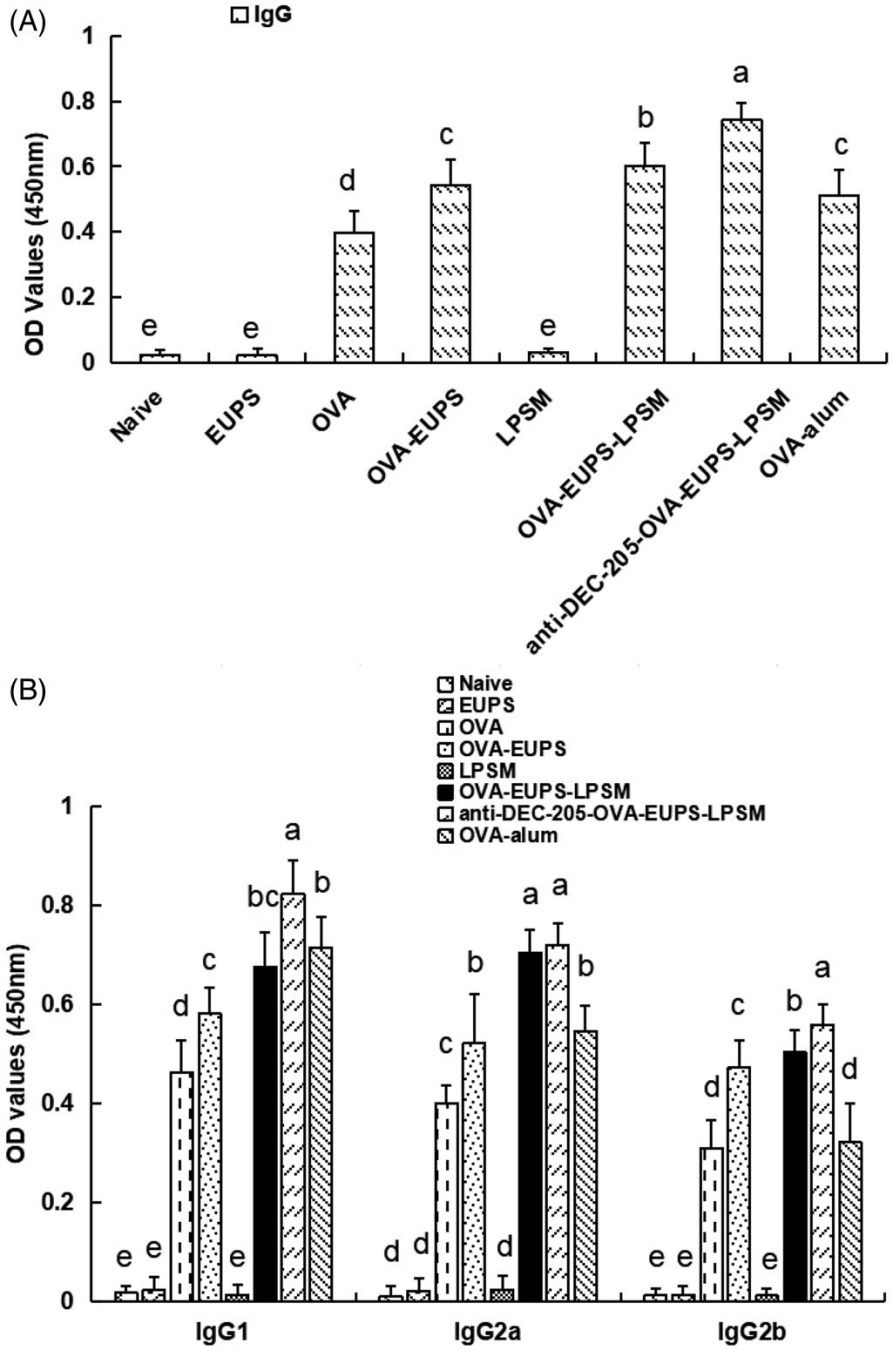
Effect of the anti-DEC-205-EUPS-OVA-LPSM nanoparticles on antibody response. Serum samples were collected for ELISA from immunized ICR mice of all groups on day 14 after the second immunization. The OVA-specific IgG (A), IgG isotopes (B) levels were determined by ELISA, as mentioned in Section 2. The IgG and IgG isotopes concentration is presented as a mean ± standard deviation. (*n* = 10). Bars marked with different letters (a–e) indicate the statistically significant differences (*p* < .05).

ELISA evaluated the effect of the anti-DEC-205-EUPS-OVA nanoparticles on the humoral immune response in mice. The blood sample of the ICR mice was collected 14 days post-second booster. The serum was separated and stored in a refrigerator at −20 °C to evaluate the antibody levels. The outcomes indicated that the antibody level of the anti-DEC-205-EUPS-OVA nanoparticles group was significantly higher than the naive, EUPS, OVA, OVA-EUPS, blank LPSM, OVA-EUPS-nanoparticles, and OVA-alum groups (Figure 8). It suggests that the anti-DEC-205-EUPS-OVA nanoparticles can significantly enhance the level of specific antibodies in the sera of immunized ICR mice. The present results demonstrate that the DEC-205 receptor-targeted nanoparticles could improve the presentation efficiency of the OVA antigen substantially.

#### 3.3.2. Effects of the anti-DEC-205-EUPS-OVA-LPSM on the splenocyte proliferation

**Figure 9.**
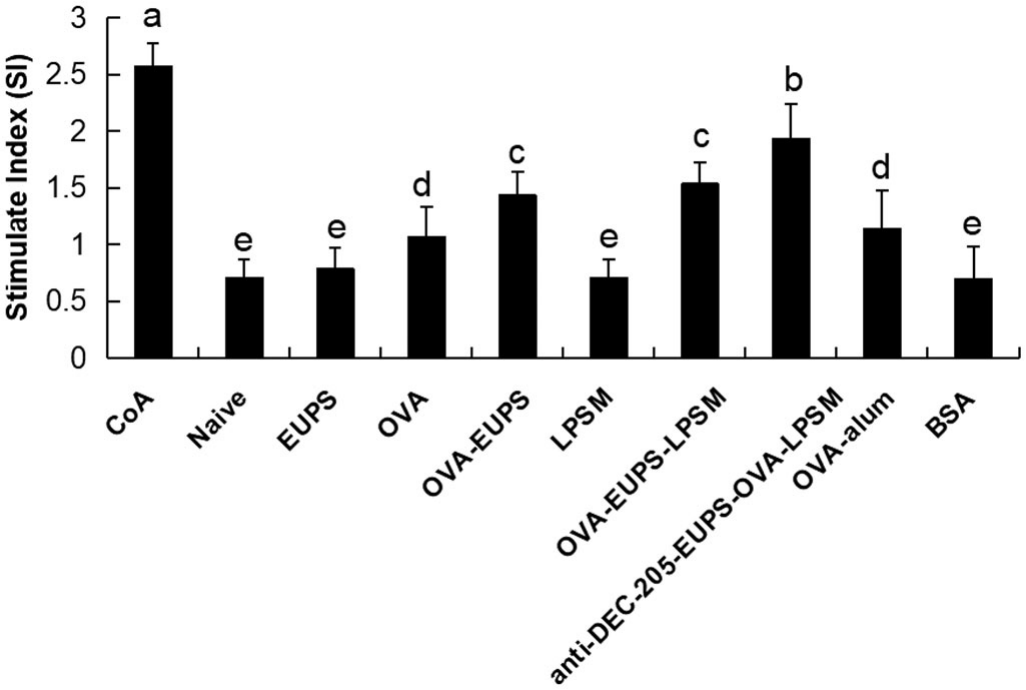
The anti-DEC-205-EUPS-OVA-LPSM nanoparticles up-regulated splenocytes proliferation *in vitro*. To investigate the anti-DEC-205-EUPS-OVA-LPSM nanoparticles on splenocyte proliferation *in vitro*, splenocytes were isolated from ICR mice in all groups on day 14 after the last immunization. CCK-8 method evaluated the splenocyte proliferation. ConA was used as a positive control, and BSA was used as a negative control. The proliferation activity was represented as stimulated index (SI). Bars marked with different letters (a–e) indicate statistically significant differences (*p* < .05) (*n* = 10).

As depicted in Figure 9, the current findings indicate that the splenocyte proliferation activity in the anti-DEC-205-EUPS-OVA-LPSM nanoparticles group was substantially escalated than the other groups (*p* < .05). In the present study, the anti-DEC-205-EUPS-OVA-LPSM nanoparticles, as a novel delivery system, demonstrated a significant ability of splenocyte proliferation as compared to the control group.

#### 3.3.3. Effects of the anti-DEC-205-EUPS-OVA-LPSM on the T-lymphocyte subset levels in the peripheral blood of immunized mice

**Figure 10.**
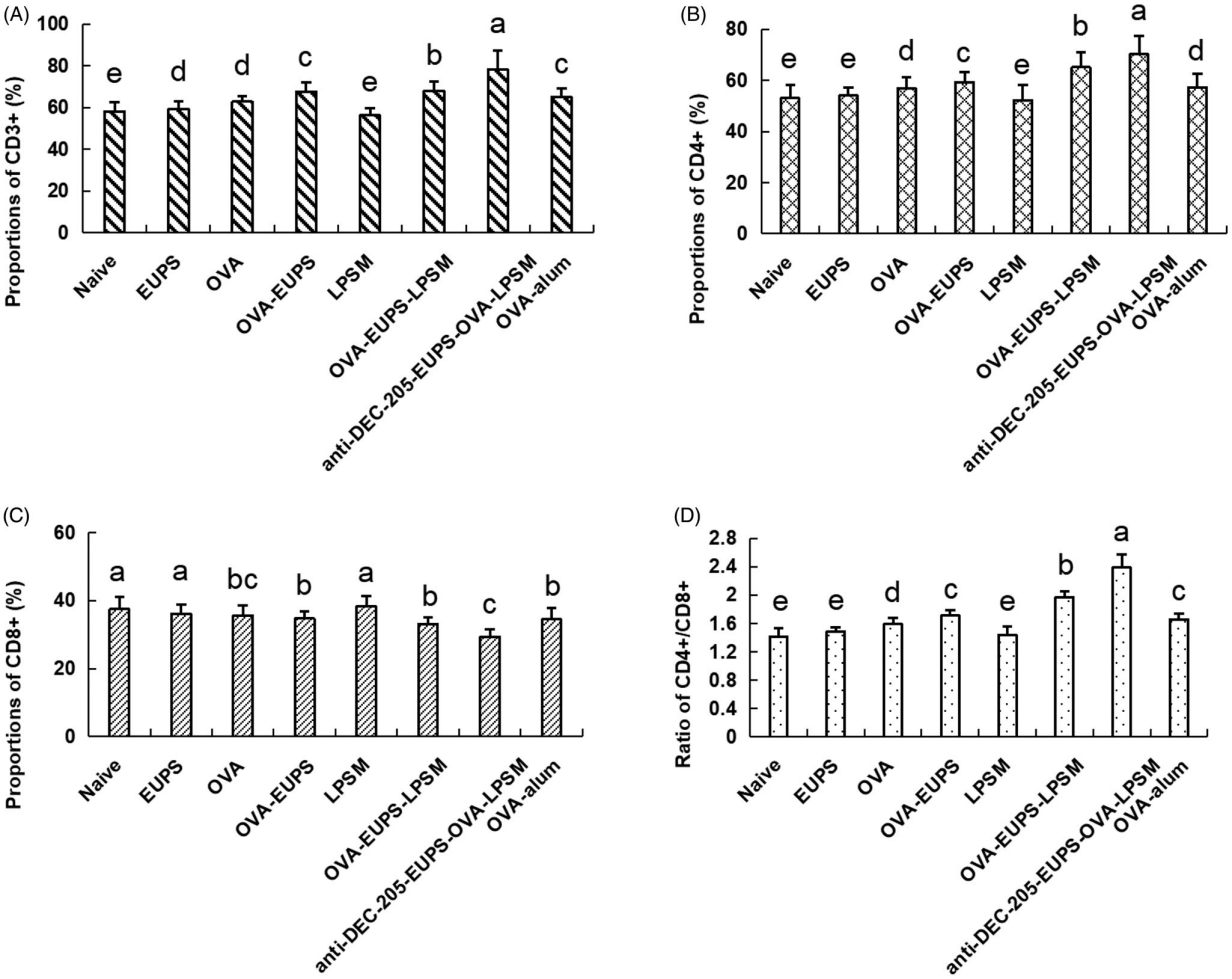
Effects of the anti-DEC-205-EUPS-OVA-LPSM on peripheral blood CD3^+^, CD4^+^, CD8^+^ cells, and CD4^+^ to CD8^+^ ratio in mice. 14 days after the second immunization, blood samples were collected, and (A) CD3^+^, (B) CD4^+^, (C) CD8^+^ cells, and (D) CD4^+^/CD8^+^ ratio was evaluated by FACS. Results are presented as the mean ± SD (*n* = 4), Bars marked with different letters (a–e) indicate the statistically significant differences (*p* < .05).

T lymphocyte subsets serve a significant role in immune responses (Malin et al., 2020; Sánchez et al., 2020). The CD3^+^ antigen serves as a T-lymphocytes biomarker. T lymphocytes can be categorized as T helper lymphocytes and inhibitor lymphocytes (Bellesoeur et al., 2020). The CD4^+^ antigen serves as a T helper lymphocyte marker, and it also induces antibody secretion from B cells. The CD8+ antigen, a T lymphocyte surface marker, can reflect cellular immune response. In an enhanced humoral immune response, the CD4^+^ cells count increases, and CD8^+^ cell count decreases, whereas the CD4^+^ to CD8^+^ ratio increases (Piris et al., 2020). Thus, the CD4^+^ to CD8^+^ ratio regulates the activation and inhibition of the immune system (Arroyo & Pello, 2020; He et al., 2020). In the current study, as depicted in Figure 10, the frequency of CD3+ and CD4^+^ T lymphocytes and the CD4^+^ to CD8^+^ ratio increased substantially in the anti-DEC-205-EUPS-OVA-LPSM treated group as compared to the other groups. The CD3^+^ and CD4^+^ T lymphocytes counts, and the CD4^+^ to CD8^+^ ratio in the EUPS-OVA-LPSM groups was remarkably higher than the EUPS-OVA, EUPS, LPSM, OVA, EUPS-alum, and naive groups. The results in the EUPS-OVA group was significantly higher than the OVA, EUPS, LPSM, EUPS-alum, and naive groups. Conversely, the CD8+ T lymphocytes level in the anti-DEC-205-EUPS-OVA-LPSM groups was considerably lower than the other groups. The data revealed that both the anti-DEC-205-EUPS-OVA-LPSM group and the EUPS-OVA-LPSM group could partially promote the immune response by selectively increasing the T cell subsets in immunized mice. The anti-DEC-205-EUPS-OVA-LPSM nanoliposomes demonstrated a more significant effect than the EUPS-OVA-LPSM. This suggests that the DEC-205 receptor-targeted nanoliposome can enhance the efficacy of EUPS-OVA-LPSM delivery to the T cell subsets.

#### 3.3.4. Effects of the anti-DEC-205-EUPS-OVA-LPSM on serum cytokine concentration in mice

**Figure 11.**
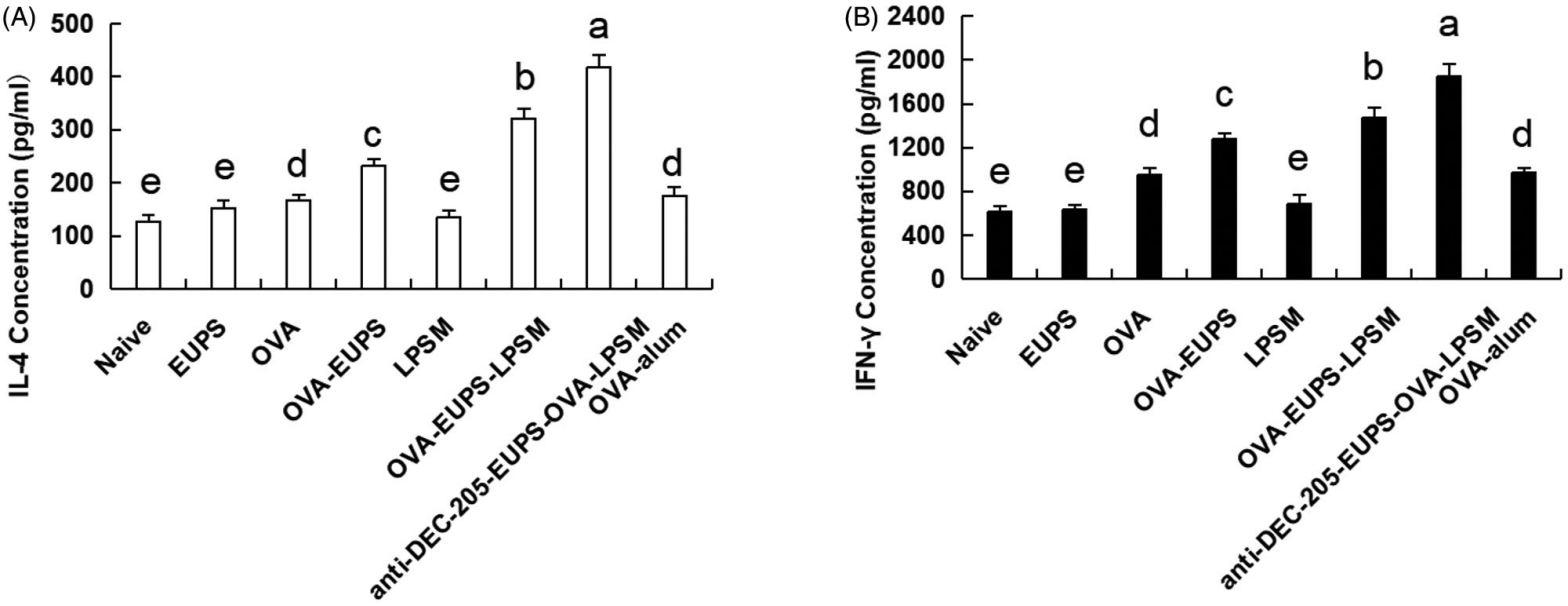
The **a**nti-DEC-205-EUPS-OVA-LPSM effect on the serum cytokines concentration of mice. 14 days after the second immunization, serum samples were collected, and the (A) IL-4 and (B) IFN-γ levels were determined by ELISA. Results are presented as the mean ± SD (*n* = 4), *p* < .05. Bars marked with different letters (a-e) indicate statistically significant differences.

Cytokine levels act as the immune response biomarker in animals. Cytokines are crucial proteins with an ability to regulate the T helper cells function (Imanishi & Saito, 2020). Typically, Th1-cytokines such as IFN-γ, IL-2 can promote lymphocytes division and proliferation, DC maturation, and activate Th1-based immune responses (Zivcec et al., 2020). Th2 cytokines, such as IL-4, −6, and −10, promote Th2-type immune responses and enhance antibody production (Chilakapati et al., 2020; Passos et al., 2020). The findings of the current study revealed that the IL-4 and IFN-γ concentrations in the anti-DEC-205-EUPS-OVA-LPSM group were notably greater than the other groups. Additionally, the IL-4 and IFN-γ cytokine concentration in the EUPS-OVA-LPSM and the EUPS-OVA groups were escalated remarkably (*p* < .05; Figure 11) than the OVA, EUPS, LPSM, EUPS-alum, and naive groups. It showed that the anti-DEC-205-EUPS-OVA-LPSM nanoliposomes significantly escalated the Th1 and Th2 cytokines production in the ICR mice, which suggests that the anti-DEC-205-EUPS-OVA-LPSM can encourage the Th1 and Th2 responses simultaneously. The anti-DEC-205-EUPS-OVA-LPSM led to enhanced IL-4 and IFN-γ cytokine production than the EUPS-OVA-LPSM and EUPS-OVA, which indicate that the EUPS-OVA nanoliposome significantly potentiated the immunological activity of EUPS-OVA. It also suggests that the DEC-205 receptor-targeted nanoliposomes can enhance the efficacy of EUPS-OVA-LPSM delivery in mice. These results demonstrate that the anti-DEC-205-EUPS-OVA-LPSM can substantially stimulate the Th cells to secrete the Th1- and Th2-type cytokines.

#### 3.3.5. Effects of anti-DEC-205-EUPS-OVA-LPSM on cytotoxic T lymphocyte (CTL)

**Figure 12.**
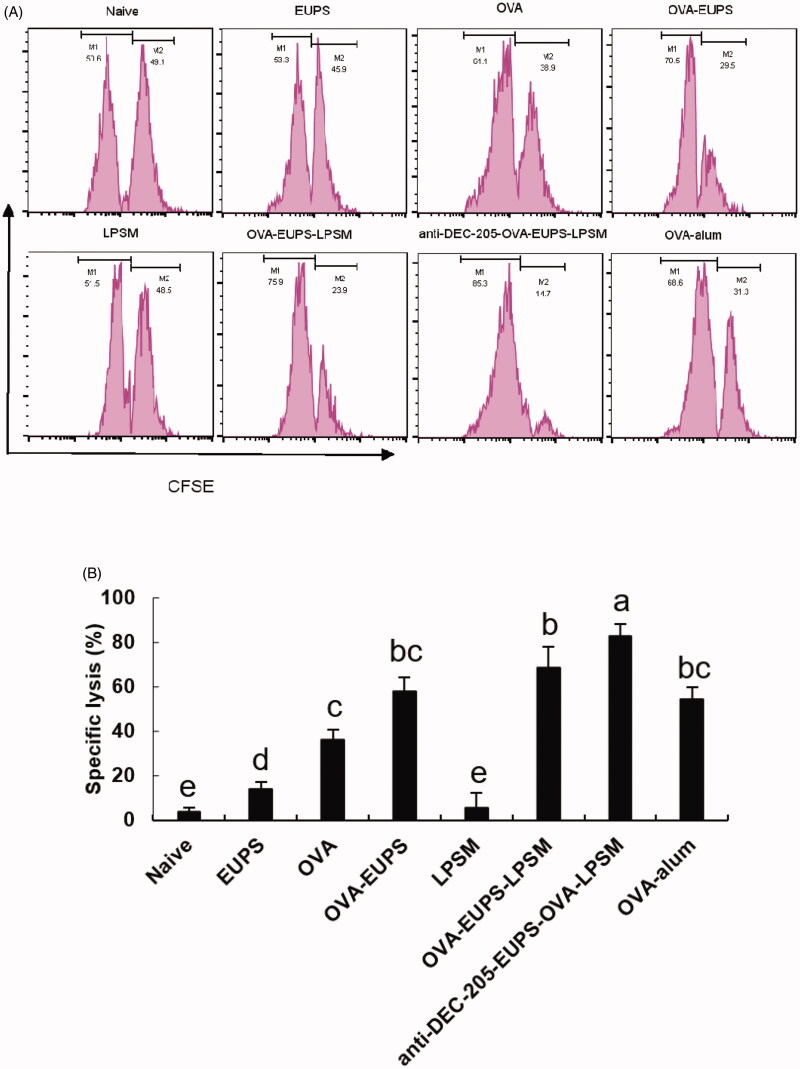
(A,B) The anti-DEC-205-EUPS-OVA-LPSM nanoparticles enhanced cytotoxicity *in vivo*. The anti-DEC-205-EUPS-OVA-LPSM nanoparticles effects on the OVA-specific cytotoxicity was evaluated *in vivo*; CFSE labeled target cells (CFSE^high^ and CFSE^low^ at 1:1 ratio) were prepared and injected intravenously to OVA-immunized mice on day 14 after the second immunization. Four hours later, the mice were sacrificed, spleens were harvested, and single-cell suspensions of splenocytes were prepared. The altered CFSE^low^ (M1) and CFSE^high^ (M2) target cell population ratio was evaluated. The percentage of OVA-specific lysis is depicted in Figure 7(A,B). The lysis percentage was determined for each group, as mentioned in Section 2. Bars marked with different letters (a–e) indicate statistically significant differences (*p* < .05) (*n* = 4).

CTL cell killing activity is a crucial immune defense process, which reflects the level of the cellular immune response. The killing ability of CTL was measured on the 14th day post-second immunization to investigate the effect of the anti-DEC-205-EUPS-OVA nanoparticles on specific CTL responses. The OVA-specific killing target cells percentage was 82.8% in the anti-DEC-205-EUPS-OVA-LPSM nanoparticles group mice. The killing percentage of the naive, EUPS, OVA, OVA-EUPS, LPSM, OVA-EUPS-LPSM, and the OVA alum groups were 2.9, 13.8, 36.3, 58.2, 5.8, 68.5, and 54.3%, respectively. The results showed that CTL killing activity in the anti-DEC-205-EUPS-OVA-LPSM nanoparticle group was significantly higher than the naive, EUPS, OVA, OVA-EUPS, LPSM, OVA-EUPS-LPSM, and OVA-alum groups. The preliminary results revealed that the anti-DEC-205-EUPS-OVA nanoparticles could substantially increase the OVA-specific CTL killing activity (Figure 12).

#### 3.3.6. Effect of the anti-DEC-205-EUPS-OVA nanoparticles on the NK cell activity

**Figure 13.**
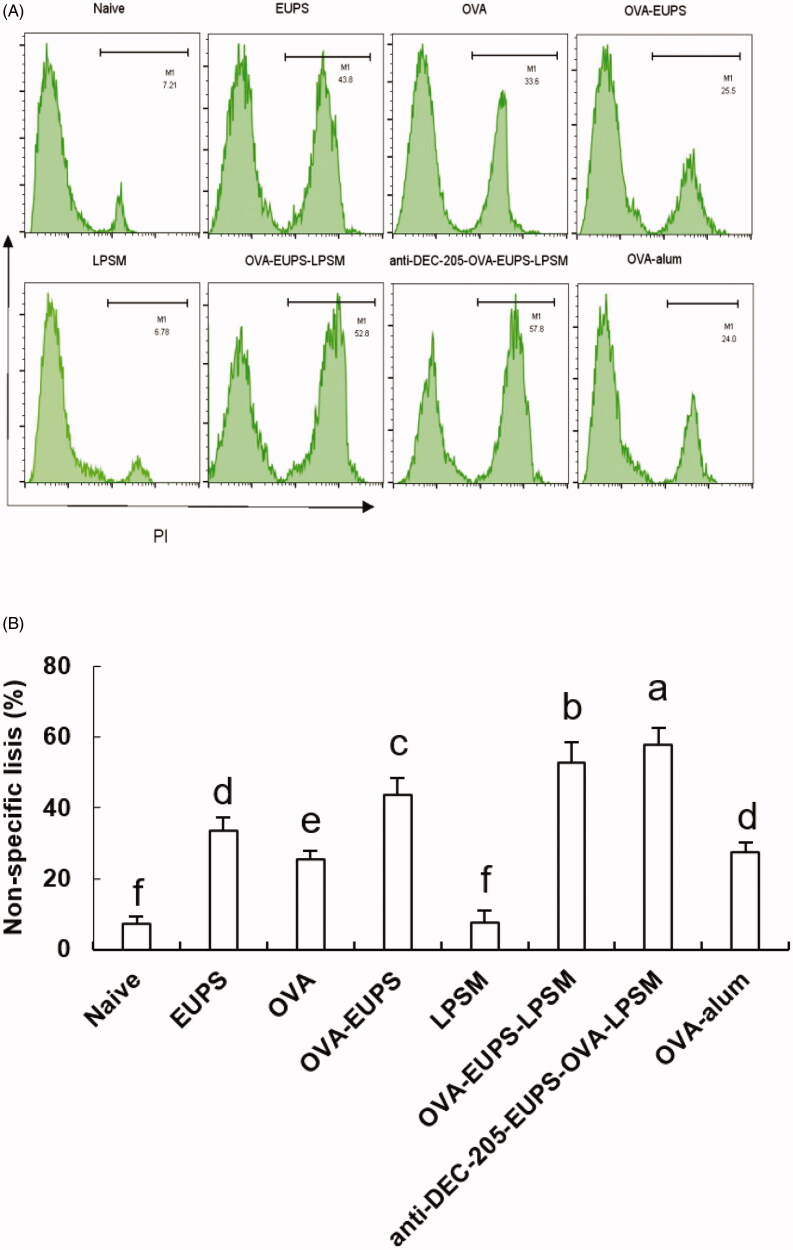
(A, B) The **a**nti-DEC-205-EUPS-OVA-LPSM nanoparticles increased the killing activity of NK cells. To examine the anti-DEC-205-EUPS-OVA-LPSM nanoparticles effect on the NK cells killing activity, *in vitro*, K562 cells labeled with CFSE were employed as target cells while splenocytes from immunized mice in all groups were employed as effector cells. After killing 4 h, the PI was added to the reaction mixture to stain dead cells. Later the killing percentage of NK cells was analyzed by FACS. The CFSE and PI double-positive cells were selected and recorded. The percentage of CFSE and PI double-stained cells in total CFSE positive cells represent the nonspecific lysis percentage, as demonstrated in Figure 4(A,B). The lysis percentage of NK cells was calculated for each group, as described in the experimental section. Bars marked with different letters (a–e) indicate statistically significant differences (*p* < .05) (*n* = 4).

It is well known that the killing activity of NK cells is a nonspecific protection process of the immune system. In the current study, we designed experiments in which FACS was used to determine the NK cell killing activity of the anti-DEC-205-EUPS-OVA-LPSM nanoparticles cells against the target cells (K562 cells) in mice. We found that the killing percentage of NK cells in mice in the anti-DEC-205-EUPS-OVA-LPSM nanoparticles group and the OVA-EUPS-LPSM group was 57.8% and 52.8%, respectively. The killing percentages in the naive, EUPS, OVA, OVA-EUPS, LPSM, and OVA-alum groups were 7.21, 33.6, 25.5, 43.7, 7.55, and 27.6%, respectively. These findings showed that the killing activity of NK cells in the anti-DEC-205-EUPS-OVA-LPSM nanoparticles immunized mice was significantly enhanced as compared to the other groups. Also, the NK cells killing activity in the OVA-EUPS-LPSM group was substantially greater than the naive, OVA-EUPS, EUPS, OVA, LPSM, and OVA-alum groups. The Eucommia polysaccharide nanoparticles, coupled with the anti-DEC-205 antibody, showed significantly higher NK cells killing activity in mice immunized with *E. ulmoides* Oliv. as compared to other groups. Thus, it suggests that the anti-DEC-205 targeting polysaccharide nanoparticles notably enhanced the NK cells killing activity (Figure 13(A,B)).

#### 3.3.7. The effect of the anti-DEC-205-EUPS-OVA-LPSM nanoparticles on the maturation of dendritic cells (DCs) in vivo

**Figure 14.**
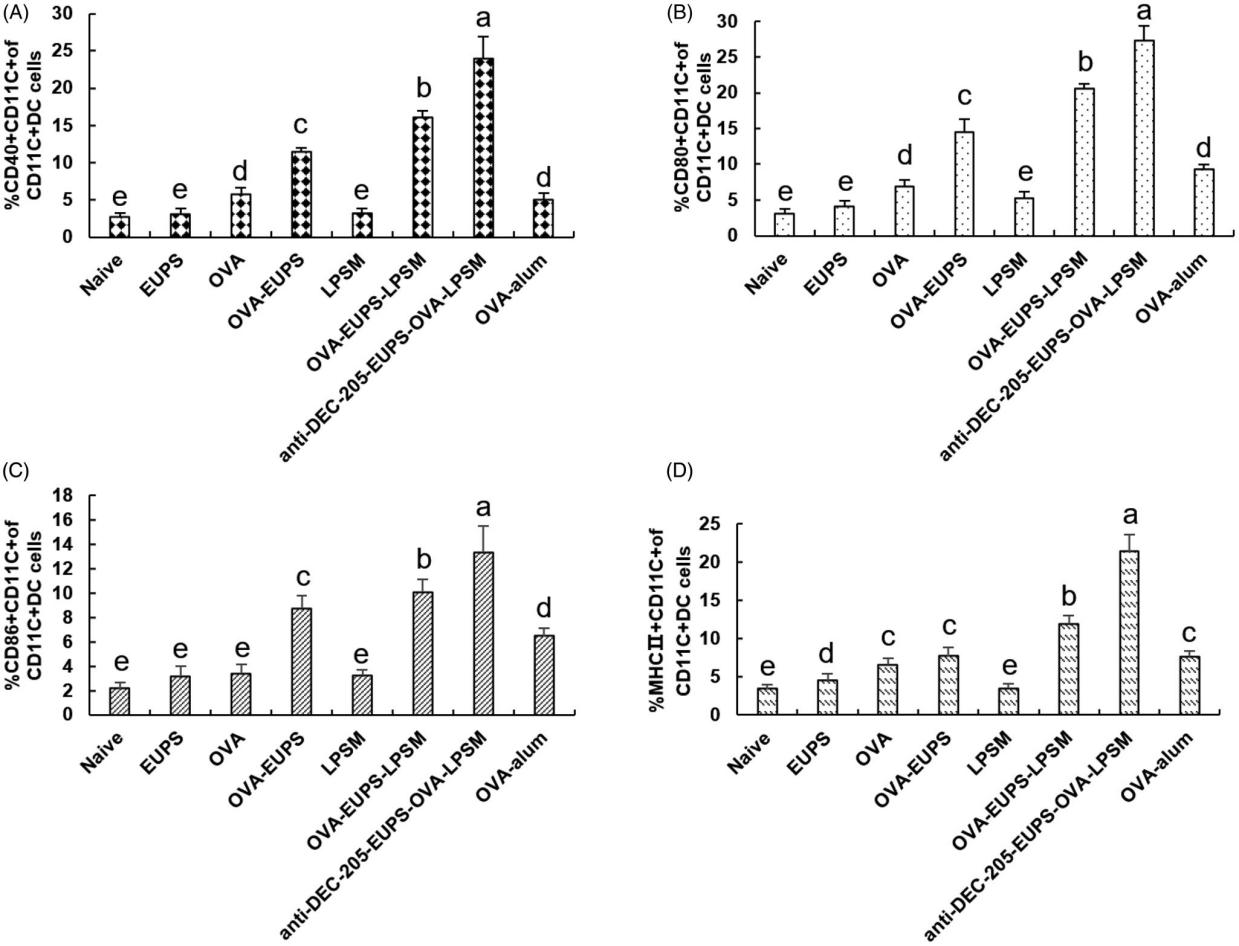
Effect of the anti-DEC-205-EUPS-OVA-LPSM on the DCs maturation. The spleens were removed from immunized mice on day 3 after the first immunization, and the splenocytes suspension was prepared. The cells were double-stained with anti-CD11c-FITC and anti-MHC II-PE, anti-CD11c-FITC and anti-CD80-PE, anti-CD11c-FITC and anti-CD86-PE, or anti-CD11c-FITC and anti-CD40-PE. The expression levels of surface molecules (MHC II, CD40^+^, CD80^+^, and CD86^+^) were analyzed using FACS. FITC stained positive cells represented the DC cells. PE stained cells represented the cells expressing surface molecules. The PE and FITC double-positive cells represented the DCs expressing surface molecules. The percentage of PE and FITC double-positive cells in total cells was calculated and recorded for each sample. The percentage of MHC II, CD40^+^, CD80^+^, and CD86^+^ in total DCs are shown in (A–D). The values are presented as mean ± standard deviation (*n* = 5). Bars marked with different letters (a–e) indicate statistically significant differences (*p* < .05).

The MHC II, CD40+, CD86+, and CD80+ are the crucial bio-markers for the maturated DCs, and the up-regulation of these molecules indicates the maturity level of DCs. The MHC II, CD40+, CD86+, CD80+ expression levels were determined on day 3 after the first immunization to investigate the effect of anti-DEC-205-EUPS-OVA-LPSM nanoparticles on the DCs maturation in mice. The MHC II, CD40+, CD80+, CD86+ expression level in the ?? nanoparticles group was found to be substantially higher than the other groups (Figure 14). The expression of surface molecules in the anti-DEC-205-EUPS-OVA-LPSM nanoparticles group and the OVA-EUPS-LPSM group was considerably higher than the naive, EUPS, OVA, OVA-EUPS-LPSM, and LPSM groups. The results showed that the effect of polysaccharide nanoparticles on the maturation and antigen presentation of DCs was significantly higher than the non-targeted nanoparticles. Additionally, the anti-DEC-205-targeting polysaccharide nanoparticles demonstrated a more significant effect on activating and inducing the DC maturation. Its targeting effect needs to be further verified, and its specific molecular mechanism needs to be explored.

## 4. Discussion

The particle size is a crucial characteristic to evaluate the stability of liposome (Dutta et al., 2020). In the present study, the colloidal stability of anti-DEC-20-EUPS-OVA-LPSM was evaluated by determining the particle size at 25 °C and 4 °C for 14 days. As illustrated in Figure 1(A,B), the particle size of anti-DEC-20-EUPS-OVA-LPSM gradually decreased with an increase in storage time at 4 °C or 25 °C. Moreover, the particle size of liposomes stored at 25 °C reduced significantly than the liposomes stored at 4 °C. The present data revealed that anti-DEC-20-EUPS-OVA-LPSM remained stable and showed high integrity at 4 °C. However, the leakage rate increased significantly at 25 °C (*p* < .05), which was related to the increase of membrane fluidity and the decrease of the stability of liposome coalescence. The antibody on the surface of immunoliposome may also increase the interaction between liposomes and reduce the stability of the liposome membrane (Dutta et al., 2020). Therefore, the liposomes should be preserved at low temperature (4 °C).

Free antigens lead to immune tolerance rather than immune response. Depending upon the size, microspheres, nanoparticles, liposomes, or other microparticles, antigen-carriers targeted toward DCs can get easily absorbed by the DC as compared to the free antigens where cross-antigen presentation plays a crucial role in preventing the antigen degradation (Sharma et al., 2015). Liposomes are a type of soft spherical lipid bilayer vesicles, primarily contains phospholipids and cholesterol, and possess hydrophobic and hydrophilic regions (Bangham et al., 1965). Their formation is chiefly driven by hydrophobic interactions and other intermolecular forces (Marsh, 2012; Zahednezhad et al., 2019). Nanoliposome, as a novel drug carrier demonstrates the following characteristics: (1) enhances the specificity of drugs toward specific sites; (2) extends the time of drug action as it leads to slow drug release; (3) reduces the drug dosage, and either decrease or prevent the side effects of drugs; (4) increases the stability of biomacromolecule drugs such as peptides and proteins. Antigen-loaded liposomes elicit both antigen-specific humoral and cellular immune responses (Sharma et al., 2015). It is commonly used either as a vaccine delivery system or as an adjuvant with distinct antigens. It has a higher delivery efficiency than alum or Freund’s adjuvant, as it specifically targets DCs (Luo et al., 2017; Tang et al., 2020). Antigen loaded nanoparticles, along with synthetic biodegradable materials are used to target the DCs.

DEC-205 receptor, also known as CD205, is a type I membrane protein and belongs to the C-type lectin superfamily. It has a relative molecular weight of 205 kD and 10 sugar recognition domains (Iberg & Hawiger, 2019; Padilla-Quirarte et al., 2019; Zahednezhad et al., 2019). Multiple studies have demonstrated that the vaccine targeted at the DEC-205 receptor could deliver the antigen and drug to DCs more efficiently to induce a significant immune response and protective immunity (Do et al., 2012). Badiee et al. (2007) constructed the anti-human DEC-205 immunoliposomes (anti-hDEC-205 iLPSM) and evaluated the delivery efficiency of targeted nanoliposomes to DCs (MoDC or CD1c^+^ BDC), in vitro. The outcomes demonstrated that the antibody conjugated DEC-205 enhanced the immature and mature MoDCcounts. Besides, CD1c^+^ BDC phagocytosing anti-hDEC-205 iLPSM and anti-hDEC-205 iLPSM were phagocytosed and made available for antigen processing by DC. It demonstrated that DEC-205 is an effective target receptor for delivering liposomes to human DCs (Badiee et al., 2007). Cruz et al. (2014) designed a PEGylated Poly(lactic-co-glycolic acid) (PLGA) nanoparticles encapsulated ovalbumin, which was targeted at the CD40, a TNF-α family receptor, DEC-205, a C-type lectin receptor, and CD11c by using specific monoclonal antibodies (mAbs) conjugated to the NP. The result demonstrated targeted NP with higher efficacy than the non-targeted NP in stimulating CD8 + T cell responses. It revealed that the delivery of NP-vaccines to DC by specifically targeting DC’s cell-surface molecules enhanced the vaccine potency and induction of T cell responses than the nonspecific delivery of NP to DC (Cruz et al., 2014). In our study, the level of OVA-specific antibody titer, cytokine concentration, splenocyte proliferation SI, and NK cell and CTL killing activity in the anti-DEC-205-EUPS-OVA-LPSM nanoliposomes group was significantly higher as compared to the OVA-EUPS-LPSM, OVA-EUPS, OVA-alum, and OVA groups. This suggested that the DEC-205 receptor-targeted nanoliposomes significantly increased the non-targeted liposome specific humoral and cellular immune response. The DC’s maturation level, surface molecules biomarkers (MHC II, CD40^+^, CD80^+^, and CD86^+^) expression level in the anti-DEC-205-EUPS-OVA-LPSM nanoliposomes group was significantly higher than the OVA-EUPS-LPSM, OVA-EUPS, OVA-alum, and OVA groups. This suggested that the DEC-205 receptor-targeted nanoliposomes significantly facilitated the DC maturation. These findings revealed that the anti-DEC-205-EUPS-OVA-LPSM promoted the immune response by targeting the DEC-205 receptor, which in turn increased the polysaccharide and OVA delivery efficiency.

Certain natural polymers, which are used to design DC targeted nanoparticles, have strong immunoenhancement properties along with good safety and low toxicity (Liu ing et al., 2016). Bo et al. have shown that nanoliposomes containing *Lycium barbarum* polysaccharides (LBP) can serve as an efficacious immune adjuvant to enhance immune responses. Their data showed that the liposomes containing OVA and LBP (LBPL-OVA) could effectively enhance the antigen specificity and further promote antibody production as well as CD4^+^ and CD8^+^ T cell proliferation *in vivo*. These results revealed that LBP (LBPL-OVA) elicited a significant humoral and cellular immune response (Bo et al., 2017). Huang et al. synthesized a liposome adjuvant containing *Rehmannia glutinosa* polysaccharide liposome (RGPL), and its effect on the RGPL-induced immune response was evaluated. The outcome of this study demonstrated that the RGPL up-regulated the splenic lymphocyte proliferation level and OVA-specific IgG titers of ovalbumin-RGPL-vaccinated mice. It also increased the expression level of MHC II^+^ CD11c^+^ and CD86^+^ CD11c^+^ cells in the draining lymph nodes, central memory cells (TCM), and effector memory cells (TEM). RGPL could facilitate sufficient antigen exposure in lymph nodes. It suggests that RGPL could enhance dendritic cell maturation and functions (Huang et al., 2016). In our previous study, we found that the EUPS as an adjuvant can induce DCs maturation and enhance the antigen-specific immune response in vivo and in vitro. In the present study, the level of OVA-specific antibody titer, cytokine, splenocyte proliferation, and NK cell and CTL cytotoxicity in the anti-DEC-205-EUPS-OVA-LPSM nanoparticles and OVA-EUPS-LPSM group was substantially greater than the EUPS and OVA group. It suggests that the EUPS encapsulated within the liposomes with OVA act as a DCs maturation modulator and immune enhancer. Besides, it significantly promoted the expression of DCs maturation biomarkers (CD40^+^, CD80^+^, and CD86^+^), MHC II, and OVA-specific humoral and cellular immune responses.

Th1 or Th2 responses can be regulated by antigenic stimulation and T-cell cytokines to prevent antigen entry into the host cells (Gandhi et al., 2018; Mahlangu et al., 2020). Th1 cytokines secreted by the Th1 cells can stimulate the DCs maturation and increase the lymphocyte proliferation and division to induce cell-mediated immunity. Th2 cytokines secreted by Th2 cells can elicit humoral immune responses (Gandhi et al., 2017). An ideal vaccine can elicit balanced Th1/Th2 responses to induce protective immunity against a certain infectious antigen. In a study by Bandyopadhyay et al. (2011), DCs were incubated with the nanoparticles or s-OVA-DEC-205 conjugated nanoparticles for 8 h. Targeted nanoparticles induced cytokines production from DC to promote the Th1 and Th2 cell cytokine production (Bandyopadhyay et al., 2011). In the current study, we evaluated the expression of Th1/Th2 cytokines in all groups of mice. Our data indicated that the anti-DEC-205-EUPS-OVA-LPSM nanoparticles significantly increased the IFN-γ and IL-4 concentration in the serum (Figure 7). Additionally, the anti-DEC-205-EUPS-OVA-LPSM can remarkably up-regulate the IgG isotypes (IgG1, Ig2a, and IgG2b). The present data suggest that the DEC-205 mediated DCs-targeted nanoliposome promoted the Th1 and Th2 immune responses simultaneously.

The host elicits both cellular and humoral immune response against an antigen (Sun et al., 2018). The cell-mediated immune response is a crucial part of protective immunity, which regulates the T cells and destroy and clear the intracellular microbes. The humoral immune response leads to antigen-specific antibody-mediated neutralization and elimination of extracellular microbes and microbial toxins (Burchill et al., 2013). In the current investigation, we observed that the anti-DEC-205-EUPS-OVA-LPSM could enhance the antibody titer of IgG and IgG isotype. These findings from the T-lymphocyte proliferation assay demonstrated that the anti-DEC-205-EUPS-OVA-LPSM could increase the splenocyte proliferation response in the immunized mice. Also, the anti-DEC-205-EUPS-OVA-LPSM could up-regulate the humoral and cellular immune response simultaneously.

CTL and NK cells killing activity assays are widely employed to examine cellular immune response levels. NK cells and Cytotoxic lymphocytes (CTLs) are two primary lymphocytes (Schwarz et al., 2013; Shao et al., 2018). CTLs are part of the CD8^+^ subset of T cells and demonstrates the presence of T-cell receptors (TCRs). CTLs can identify and eliminate pathogen-infected cells. NK cells primarily participate in the innate immune response, and the NK cells mediated eradication of pathogens and tumor cells are nonspecific. They exercise cell-mediated cytotoxicity against tumor cells and infected cells and play a regulatory role by cytokines and chemokines secretion. The sinusoidal regions of the liver and the red pulp of the spleen under resting conditions are enriched with NK cells (Halle et al., 2017). During infection, NK cells proliferate and mature from a resting to an effector state, and are more responsive and effective as killer cells. They also invade the lymphoid and non-lymphoid tissues to kill target cells (Sanchez-Martinez et al., 2019). In our study, the anti-DEC-205-EUPS-OVA-LPSM was employed as a delivery system targeted to DCs, which escalated the lytic activities of CTLs and NK cells in the ICR mice splenocytes (Figures 7 and 8). The findings of the current study revealed that the DEC-205 mediated DCs-targeted nanoliposome can increase antigen-specific and nonspecific killing activities against pathogens.

## 5. Conclusion

In the current study, the anti-DEC-205-EUPS-OVA-LPSM nanoparticles significantly induced the cellular and humoral immune responses by facilitating the DC maturation. This study suggests that the application of the DEC-205 receptor-targeted nanoliposomes can increase the delivery efficiency of the conventional liposomes’ dendritic cell-targeted drugs and antigens. The targeted nanoparticles can further improve the efficiency of drug delivery and antigen presentation by passive targeting of conventional liposomes. This study has created a platform for future research studies on targeted drug and antigen delivery and the prolongation of their efficacy.
